# Efficient adsorption of aromatic and aliphatic hydrocarbons by electrospun hydrophobic PTFE-NiO composite nanofiber filter mats

**DOI:** 10.1186/s11671-023-03834-4

**Published:** 2023-04-20

**Authors:** Syeda Irsa Mazhar, Attarad Ali, Trevor B. Tilly, Muhammad Hassaan Khan, Chang-Yu Wu

**Affiliations:** 1 Department of Environmental Science, International Islamic University, Female Campus, Room No. 23, Hazrat Maryam Block, H-10, Islamabad, Pakistan; 2 Department of Environmental Engineering Sciences, Engineering School of Sustainable Infrastructure & Environment, University of Florida, Gainesville, FL 32611-6450 USA; 3 Directorate of Quality Enhancement Cell (QEC), University of Baltistan Skardu, Gilgit-Baltistan (GB) Pakistan, Skardu, 16400 Pakistan; 4 Department of Bioinformatics & Biotechnology, Faculty of Life Sciences, Government College University Faisalabad (GCUF), Faisalabad, Punjab Pakistan

**Keywords:** Composite PTFE-NiO mats, Green electrospinning, Filtration

## Abstract

Aromatic and aliphatic hydrocarbons (AAHs) are comprised of a variety of gaseous chemicals that may affect human and environmental health. To remove AAHs from air, polytetrafluoroethylene-nickel oxide (PTFE-NiO) composite nanofiber filter mats (NFMs) were synthesized and characterized for their ability to effectively adsorb AAHs. The NiO-nanoparticle-doped mats were fabricated by green electrospinning of PTFE and polyvinyl alcohol (PVA) mixtures added with nickel (II) nitrate hexahydrate in the spinning solution followed by surface heat treatment. FE-SEM FTIR, Raman spectroscopy, sessile drop and Jar methods were applied as characterization techniques. The diameter of the electrospun nanofibers without NiO dopant ranged from 0.34 ± 21.61 to 0.23 ± 10.12 µm, whereas a reduction in diameter of NiO-doped nanofibers was obtained, ranging between pristine to 0.25 ± 24.12 µm and 0.12 ± 85.75 µm with heat treatment. 6% (by weight) NiO-doped PTFE composite NFMs exhibited a high water-contact angle of 120 ± 2.20 degrees; the high hydrophobicity value aided self-cleansing property of NFMs for practical applications. UV adsorption capability for heat-treated PTFE-NiO NFMs was evaluated for three AAHs, and the results showed that 6 wt% NiO adsorbed 1.41, 0.67, and 0.73 µg/mg of toluene, formaldehyde and acetone, respectively. These findings reveal the potential applicability of the prepared filter mats for capturing various AAHs from polluted air.

**Supplementary Information:**

The online version contains supplementary material available at 10.1186/s11671-023-03834-4.

## Highlights

1. Synthesis of PTFE-NiO porous composite NFMs by green electrospinning.
2. PTFE-NiO mat had increased surface roughness, hydrophobicity and enhanced self-cleansing property.
3. Surface modification with NiO dopant significantly enhanced adsorption of AAHs.

**Supplementary Information:**

The online version contains supplementary material available at 10.1186/s11671-023-03834-4.

## Introduction

Aromatic and aliphatic hydrocarbons (AAHs) are a major class of air pollutants found in many places: petroleum refineries, from volatilization of building materials, usage of automobiles, surface deterioration, detergents, pesticides and cosmetics [1, 2]. Prolong exposure to certain AAHs (e.g., benzene, formaldehyde, toluene, xylene, ethylene, styrene and acetaldehyde) poses toxicity and carcinogenic effects on human beings, animals and plants [3, 4]. Besides anthropogenic sources, natural sources of AAHs emissions can come from both terrestrial and oceanic environments [5]. Some AAHs undergo photochemical reactions with the nitrogen oxides (NOx) and sulfur dioxide (SO_2_), resulting in the formation of smog in the troposphere as well as stratospheric ozone layer depletion [6, 7]. The presence of AAHs in an enclosed environment requires effective management to keep them below the threshold level. Increase in usage of renewable energy sources in vehicles, such as electric vehicles (EV) or green energy, is one of the most effective ways to reduce AAHs in this sector [8, 9]. Other approaches, including [10] destruction and recovery technology, have been used. Regarding destruction, AAHs are decomposed into carbon dioxide and water through different chemical and biological techniques, such as condensation, thermal oxidation, catalytic oxidation, and bio-filtration. On the other hand, recovery technology includes absorption, adsorption and membrane separation [5]. Among them, adsorption is more cost effective, flexible and energy efficient than others [6]. For robust air filtration and purification, fibrous nano-membranes (NMs) are considered better due to their high specificity, small pore size, greater surface area to volume ratio, size-dependent optical, electronic & magnetic properties and increased intermolecular interactions surface area that may capture multi-pollutants [11]. Various types of NMs such as carbon-based nanoparticles, metal nanoparticles, and oxide nanoparticles have been explored as filter materials. These can also be used as a coating material for air filters to enhance their performance. For example, silver nanoparticles have been shown to have antimicrobial properties, which can be beneficial in reducing the growth of bacteria and viruses in the air. Similarly, iron oxide nanoparticles have been used as a coating material for air filters to remove volatile organic compounds (AAHs). NMs-based sensors can detect a wide range of air pollutants such as NOx, SOx, CO, and AAHs with high sensitivity and selectivity. These sensors can be integrated into air filtration systems to monitor air quality in real-time. These devices also use nanoparticles to generate reactive oxygen species (ROS) such as hydroxyl radicals, which can effectively break down air pollutants into harmless compounds. Nanoparticle air purifiers have shown high efficiency in removing PM, AAHs, and other air pollutants [3, 8, 11].

Conventional methods to fabricate fibrous metal oxide NMs include: ligand-assisted, [13] template, [14] chemical reduction [15] and magnetic field assisted non-aqueous wet chemical interactions [16]. Although these methods provide metal oxide NMs with large specific surface areas, they are not feasible to utilize in textiles industries, discouraging their application for air filtration. This emphasizes the utilization of electrospinning fabricated nanofiber mats chelated with metal oxide nanomaterials to mitigate the limitations of chelating nanomaterials in the filters [17, 18]. Electrospun nanofibers offer a promising alternative to the usage of activated carbon in the removal of AAHs through adsorption. Electrospinning is a relatively simple and effective method to produce continuous nanofibers with uniform diameters, high surface roughness, porosity, hydrophobicity and low surface energy [19, 20]. Moreover, the diameter of the electrospun fibers can be modulated by adjusting various solution parameters while its processing, for example solution conductivity, viscosity, applied voltage (AV), tip to collector distance (TCD) and flow rate (FR) [21]. This study proposes the use of a novel composite material made of hydrophobic PTFE and NiO nanofibers for efficient adsorption of AAHs. Electrospinning technique was used to produce the filter mats, which have high surface area and porosity, making them highly effective in adsorbing AAHs. Such filter mats led to a significant improvement in the adsorption capacity of AAHs compared to traditional filter materials. The research shows that composite filter mats could remove up to 98% of AAHs from the air. It was found that the adsorption process is driven by both physical and chemical interactions between the filter material and the VOC molecules. The proposed filter mats have potential applications in environmental and health fields, where AAHs are a major concern. The efficient removal of AAHs from the air is important for maintaining good air quality and reducing the risk of health problems associated with exposure to these compounds. The main drawback of electrospinning is the usage of hazardous solvents needed for the polymer spinning. However, by adopting green electrospinning method, one can avoid the application of hazardous solvents by replacing it with water instead, thereby avoiding generation of secondary pollution during the spinning process [21].

With advantages such as large specific surface area/pore size/porosity, uniform pore distribution, fast adsorption and good chemical stability [5, 6, 10], activated carbon (AC) is one of the most commonly used adsorbents for airborne contaminants such as benzene, aldehyde ketones, alcohols, and hydrocarbons. However, it also has disadvantages, including blockage of pores and internal channels by dust, low cost-effectiveness, fire hazard, adsorptive nature, premature malfunction of some component mixtures, non-suitability for wet flue gases, limited application due to its powdered nature, risk of polymerization from unsaturated hydrocarbons and additional issues related to regeneration. [22–24] It is significant to mention here porous materials (such as carbon-based materials, oxygen-containing materials, organic polymers, composites and so forth) have been investigated for improved adsorption of AAHs in terms of capacity, hydrophobic property, thermal stability and regenerability. The adsorption capacity of above-mentioned electrospun nanofibers has been enhanced by the incorporation of non-spinnable metal oxide nanomaterials that provides more active interaction to control AAHs [25].Among all, zeolite and organic polymer are considered to be the most widely adopted adsorbents for AAHs treatment estimated by the United States—Environmental Protection Agency (US EPA) [5, 18–21].

In the present research, polytetrafluoroethylene (PTFE) + polyvinyl alcohol (PVA) aqueous emulsion mixed with nickel (II) nitrate hexahydrate were used in the electrospinning process to create filter mats of PTFE nanofibers doped with nickel oxide (NiO) NMs. The PTFE is a non-spinnable polymer, requiring an assistant polymer that helps in electrospinning of PTFE solution efficiently. Polyvinyl alcohol (PVA) was selected as an assistant polymer due to the presence of following properties: soluble in water, non-toxic, excellent chemical stability, low decomposition temperature and lower cost. PVA has been used to aid spinning of PTFE polymer solutions, ultimately controlling viscosity and minimizing beading of formed nanofibers [26]. Nickel oxide (NiO) is reported to be a promising material in gas detection due to its favorable qualities including good gas sensing performance, thermal stability, cost effectiveness, ease of synthesis and tunable morphology [27–29]. Coating of polymer membranes with nanoparticles also improves its surface hydrophobicity and surface energy properties [5]. Khalil and his colleagues [30] used the electrospun NiO (NiAc/PVA precursors) composite nanofibers (NFs) for ammonia gas sensing by modifying the structure and microstructure of Ni NFs. The literature also demonstrated the synthesis and application of NiO-doped electrospun nanowires, nano-sheets, nano-sensors, nano-hollow structures, nano-webs and nano-composites for sensing ethanol, ammonia, alcohol and NO_2_. [28, 29, 31–34] The usage of PTFE nanofiber filter mats (NFMs) impregnated with NiO NMs has not been reported for AAHs filtration applications. Herein, we utilized this novel eco-friendly approach to synthesize porous PTFE-NiO composite NFMs by green electrospinning precursor PTFE-PVA for AAHs adsorption from air. The morphology, thermal and hydrophobic characteristics were evaluated of filter mat fabrication. To the best of our knowledge, we are the first to report the evaluation of PTFE-NiO NFMs for AAHs adsorption of air pollutant.

## Materials and method

### Materials

Nickel nitrate hexahydrate Ni(NO3)2.6H2O, (molecular weight (Mw): 290, Sigma-Aldrich, Germany), poly-vinyl alcohol (PVA, Mw3:35,000, degree of alcoholysis: 80%, degree of polymerization: 1700, BDH, Germany), poly-tetrafluoroethylene (PTFE 60 wt% dispersion in water, Sigma-Aldrich, Germany), formaldehyde (HPLC grade: ≥ 99.9%, Merck, USA), toluene (AR: ≥ 99.5%, Merck, USA) and acetone (HPLC grade: ≥ 99.9%, Sigma-Aldrich, Germany) were used in this study. All reagents were used as received from the suppliers.

### Electrospinning solution preparation

The blend for electrospinning the PTFE-PVA-Ni NFMs was prepared by mixing three solutions. The quantity of PTFE to PVA in each solution was fixed, and the nickel nitrate hexahydrate particle loading was varied as 2, 4 and 6 wt% in the blend. The first solution was of PVA prepared by adding 0.2 g of PVA powder in 2 mL (DI) deionized water, afterward 3 h of stirring at room temperature. The second solution was 4 mL PTFE dispersion (already in liquid form without further processing), and the third solution was prepared by dissolving nickel nitrate hexahydrate in water to create 2 mL with a concentration of 2, 4, and 6 wt%.

The three solutions were mixed in a 100 mL beaker and underwent continuous magnetic stirring for 24 h at room temperature. The (EC) electrical conductivity of the homogenized solutions was measured by a conductivity meter (Milwaukee-MW301, Hungary). Solutions preparation and processing parameters are enlisted in Table 1.

**Table 1:** Electrospinning Solution and process parameters

Parameters	Values
(1) Solution parameters	
Solution Compositions (wt%)	PTFE-PVA
	PTFE-PVA-NiO 2wt.%
	PTFE-PVA-NiO 4wt.%
	PTFE-PVA-NiO 6wt.%
Conductivity (µS/cm)	1852 ± 1.5
	1889 ± 2.5
	1894 ± 1.0
	1897 ± 1.8
(2) Process parameters	
Applied voltage	20 kV
Tip to collector Distance	12 cm
Flow rate	0.3 mL/h
Needle gauge	23SS
Temperature	25 °C
Relative Humidity	40%

### Electrospinning process

**Fig. 1:**
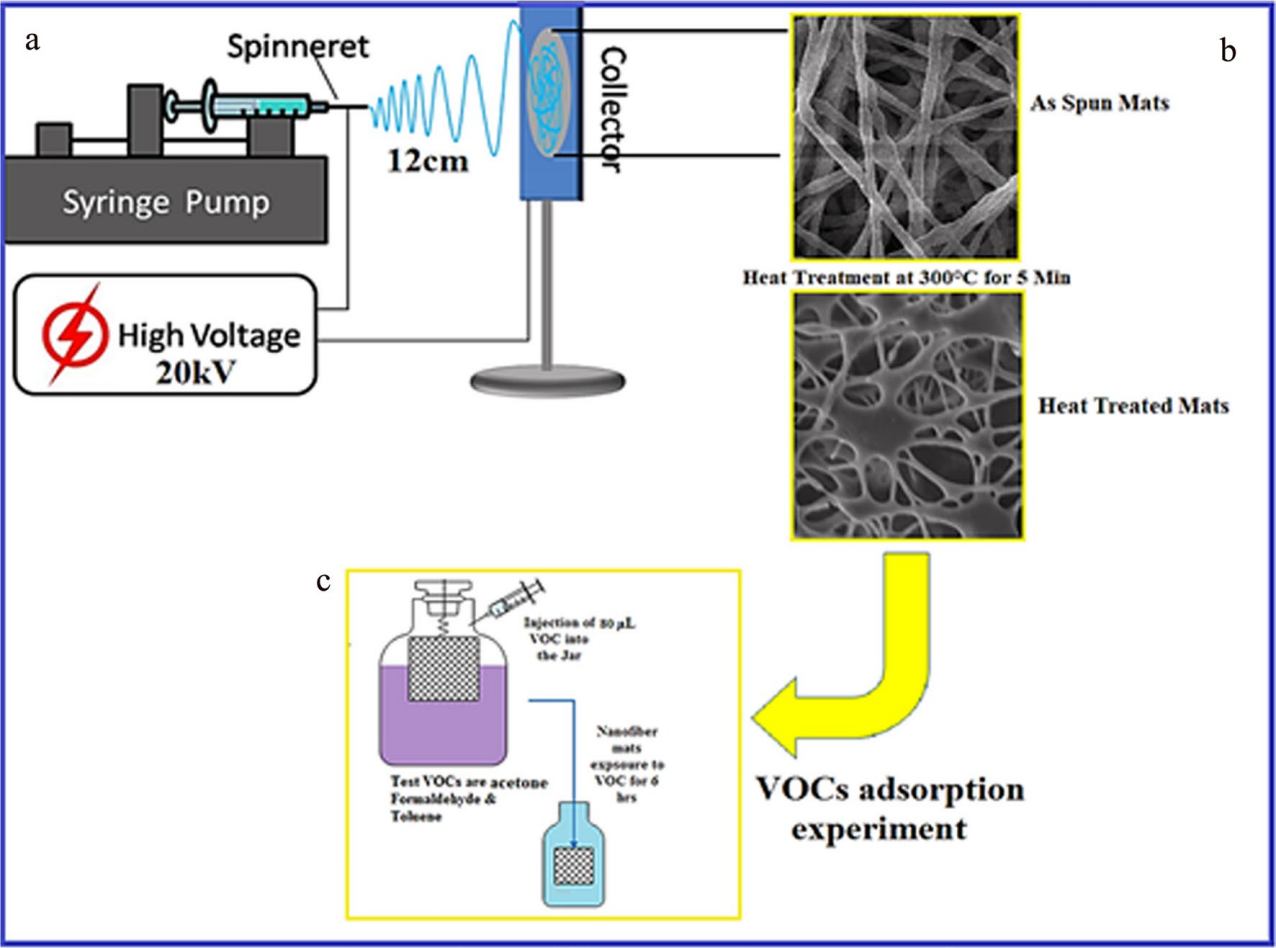
Schematic of **a** PTFE-PVA-Ni electrospinning process [16] **b** heat treatment and **c** AAHs adsorption experiment

The setup and electrospinning of the NFMs was conducted as follows. One mL of composite blend of PTFE-PVA- Ni was loaded into a disposable plastic syringe with a capacity of 5 mL, and the needle gauge was kept adjoint (23-gauge stainless steel (SS). After the syringe was carefully filled with the blend, it was fixed on a syringe pump (Parker, USA). Grounded collector in the form of a circular copper disk (diameter 20 cm) was wrapped with aluminum foil; the needle of the syringe was connected to the positive terminal of 50 kV high-voltage DC power supply (DEL Electronic Corporation, USA), and the negative terminal was connected with the collector. Detailed schematics are shown in Fig. 1. The as-prepared NFMs were dried at 60 °C for 2 h in laboratory oven and then, heated at 280 °C for 5 min in a box furnace (air atmosphere) at a heating rate of 5 °C/min, to evaporate the hydrophilic polymer carrier PVA from the precursor matrix and fuse PTFE particles. The resultant NFMs were tested after the 280 °C heat treatment, and the terminology designated as PTFE-PVA-Ni refers to the mats before heat treatment, while PTFE-NiO after full processing.

### Nanofibers characterizations

Morphology of composite NFMs was analyzed by Field Emission—Scanning Electron Microscopy (FE-SEM, JMAI/A3 TESCAN, USA). Infrared (IR) spectra of both pure and composite electrospun nanofibrous mats were obtained using an FTIR Spectrometer Nicolet 8700 equipped with a fixed 100 μm diameter aperture, and a mercury-cadmium-telluride (MCT) detector was used to analyze the absorbance in the wave number range 500–4000 cm^−1^ in the transmittance mode. Compositional analysis of the samples was performed through Laser Micro-Raman spectrometry using Dongwoo Optron’s micro-Raman spectrometer with 532 nm as an excitation laser source. The spectral range was from 100 to 2000 cm^−1^, and exposure time was 5*s*.

Water contact angle (WCA) measurement was performed with a digital camera (Sony, Cyber-shot, 5.1 megapixels) and by the static sessile water drop method [25]. All measurements were carried out at 25 °C and 45% relative humidity. A 5 μL droplet of deionized water was dropped on the surface of each electrospun NFM. The images of water drop were captured after 6*s* of addition to the surface, with at least 5 replicates. The images were processed using the drop snake analysis plugin for ImageJ software (version 1.48, Plugin: Drop Snake Analysis) [34]. Self-cleaning ability of composite NFMs was analyzed by simple dust-spreading method [25, 27].

Surface free energy (SFE) calculated based on the data acquired through water contact angle measurement using liquids having different surface tensions. The SFE mainly depends on the polymer chains organization that is directly related to the manufacturing procedures to yield films and fibers [28, 35, 36]. In this study, the obtained contact angle data on polymer NFMs surface with water were used to calculate surface free energy based on the Owens–Wendt model [35–37] with polar and dispersive contributions:

1 $$\left( {{1} + {\text{cos}}\theta } \right)\Upsilon {\text{L}} = { 2}\left( {\surd \Upsilon {\text{ld }}\Upsilon {\text{sd }} + \surd \Upsilon {\text{lp }}\Upsilon {\text{sp }}} \right)$$

The two unknowns, ϒsd and ϒsp, were calculated using the contact angle of the material tested with DI water. The surface tension values ϒL, ϒld and ϒlp for water are 72.8, 21.8 and 51.0 in mJ/m^2^. [37] Original values were obtained using Low-Bond Axisymmetric Drop Shape Analysis (LBADSA) plugin for ImageJ [35]. The LBADSA method uses a first-order approximation of the Young–Laplace equation to fit the whole image data instead of first detecting the drop contour. Porosity is often defined as the fractional empty space contained within a mat and is a morphological property independent of the characteristics of the material of the nanofibers. The porosity (*ε*) of NFMs was calculated by a standardized gravimetric method using Eq. (2) [12]

2 $$\varepsilon = p_{0} - p/p_{0} \, \times \, 100$$

VOC adsorption experiments were performed through a simple jar method as reported by Kadam [38]. Experiments were performed by hanging (with the support) the filter mats in air-tight jars for specified time duration. The adsorption study was conducted at ambient temperature and pressure using three model AAHs; formaldehyde, toluene and acetone. The selected AAHs encompass a broad range of chemical structures in both aromatic and aliphatic hydrocarbons used in a wide array of industrial applications. 80 µL (80 ppm) of each VOC was injected with the help of micropipette at the bottom of separate jars (1 L capacity), with each specimen of an area of 6.45 cm^2^ (weight around 40 mg) directly facing VOC vapors. Each jar was then sealed immediately with the lid holding the filter mats and wrapped with a para film to prevent the release of vapor. The jar was left for 6 h at room temperature (25 °C) and humidity (45%) to expose the NFMs to AAHs as shown in Fig. 1c. After the exposure, each filter mat was immersed in ethanol in an airtight glass container sealed with para film for 4 h to extract the AAHs from filter mats. AAHs extracted concentrations in the solution from nanofiber mats were systematically evaluated using a UV–Vis (Hitachi U-4001) spectrophotometer at a characteristic absorbance wavelength of 275, 287 and 300 nm. A standard calibration curve (Figure S1) with a correlation coefficient of *R*^2^ = 0.9998 was obtained for formaldehyde, 0.9977 for toluene and 0.9997 for acetone with concentrations of 0, 20, 40, 60, 80 and 100 mg L^−1^ for each of the test chemicals. The regression equation was used to convert absorbance values of AAHs expressed as AAHs adsorption per unit mass (µg/mg) of fabric sample.

## Results and discussion

### Morphology analysis

**Fig. 2:**
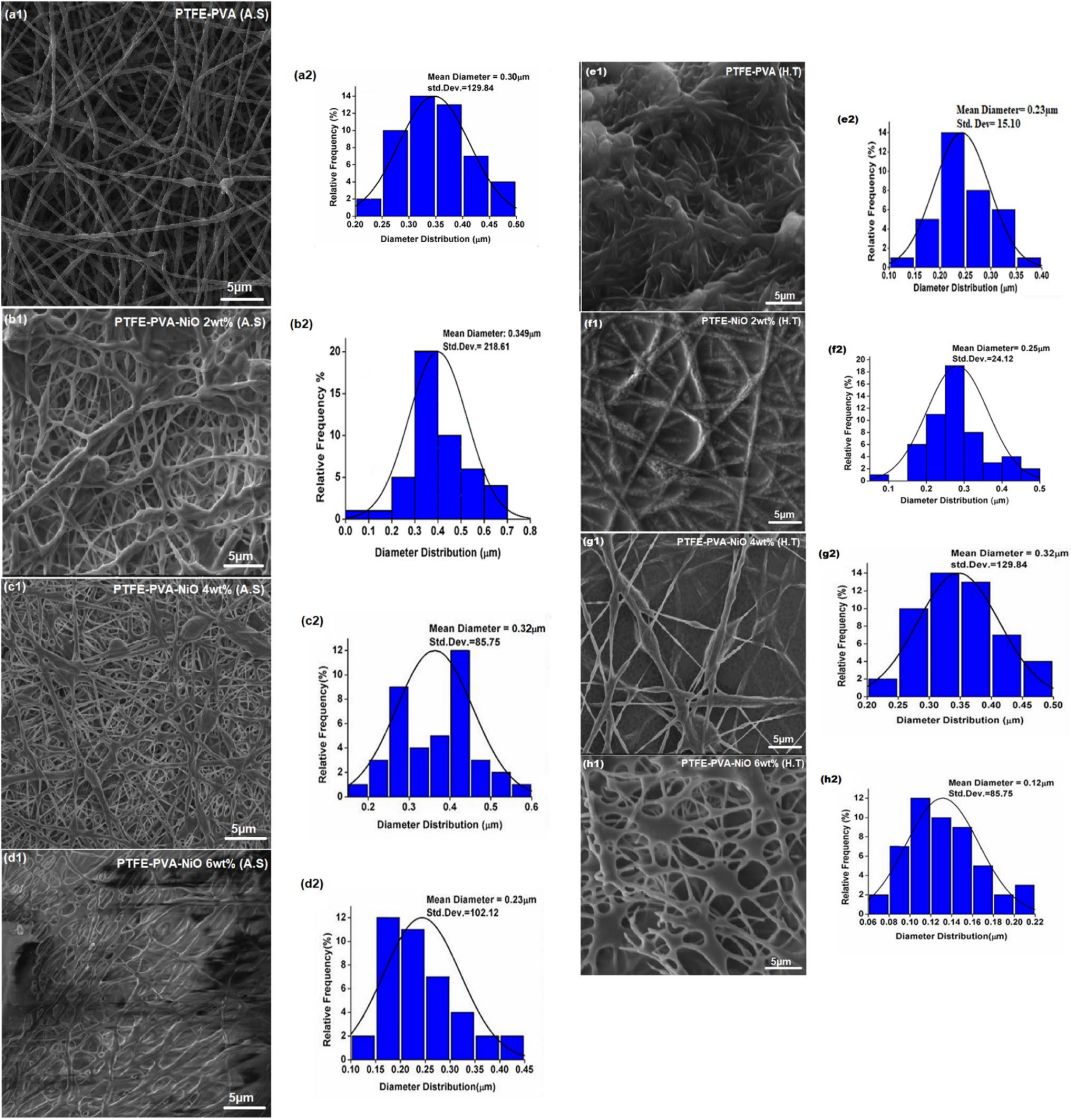
Scanning Electron Microscopy micrographs of Pure PTFE **a1**–**d1** as-spun PTFE-PVA- Ni, **e1**–**h1** and heat-treated nanofiber filter mats. **a2**–**h2** Average fiber diameter of both spun & heat-treated nano-fiber mats

The FE-SEM micrographs of composite NFMs as-prepared (PTFE-PVA-Ni) and heat-treated (PTFE-NiO) as well as their respective diameter distribution are shown in Fig. 2. Continuous fibers with porous surface morphology can be seen in SEM micrographs; however, beads were sometimes observed (see Fig. 2a1-d1), which was likely due to heterogeneity of electrospinning solutions [39]. Only one composition, PTFE-NiO 6 wt%, showed unique web shape morphology as shown in Fig. 2d1 & d2. Moreover, the mean diameter of as-prepared PTFE-PVA- Ni NFMs ranged from 0.34 ± 21.61 to 0.23 ± 10.12 µm, and their counterpart heat-treated PTFE-NiO ranged from 0.23 ± 15.10 to 0.12 ± 85.75 µm. The mean fiber diameter first increased and then decreased in case of PTFE-NiO composite nanofibers, as shown in Figure S2, due to two possible reasons: (1) increase in the applied voltage to the electrospinning solutions as shown in Figure S2a, and (2) increase in salt concentration leads to increased electrospun solution conductivity, which causes the jet to experience greater electrostatic force, eventually leading to decreased (NF) nanofiber diameter [33, 40, 41].

According to the theory of ‘electrohydrodynamics (EHD) [40], wherein the jet emerging from a solution, the jet diameter has a direct relationship with its surface tension and an inverse relationship with its solution conductivity. As the concentration of salt increases, both electrospun solution conductivity and viscosity increase at the same time. Since viscosity and surface tension are directly related for a Newtonian fluid, an increased surface tension increases the terminal jet diameter and hence, the nanofiber diameter. If a suitable dopant is present in the solution or the solution itself is very conductive due to the presence of specific type of salt like nickel nitrate hexahydrate as in present case, there will be an increase in the solution conductivity that could overwhelm the surface tension effect, leading to eventual decrease in nanofiber diameter [41]. It is thus evident that final nanofiber diameter is controlled by the competing actions of both surface tension and the net solution conductivity as shown in Figure S2. Applied voltage was optimized to 20 kV for the other compositions after measuring mean fiber diameter as shown in Figure S2a.

After heat treatment, fiber diameter was reduced due to densification of grains/nanoparticles in the PTFE fibers, complete degradation of the organic phase, and release of water vapors that had been suspended in the fiber texture. The unique porous morphology of NFMs was preserved after heat-treatment as shown in Fig. 2. Energy-dispersive spectrum of PTFE-NiO composite NFMs confirms the presence of nickel particles in the fibers as depicted in Figure S2b.

### Surface property analysis

**Fig. 3:**
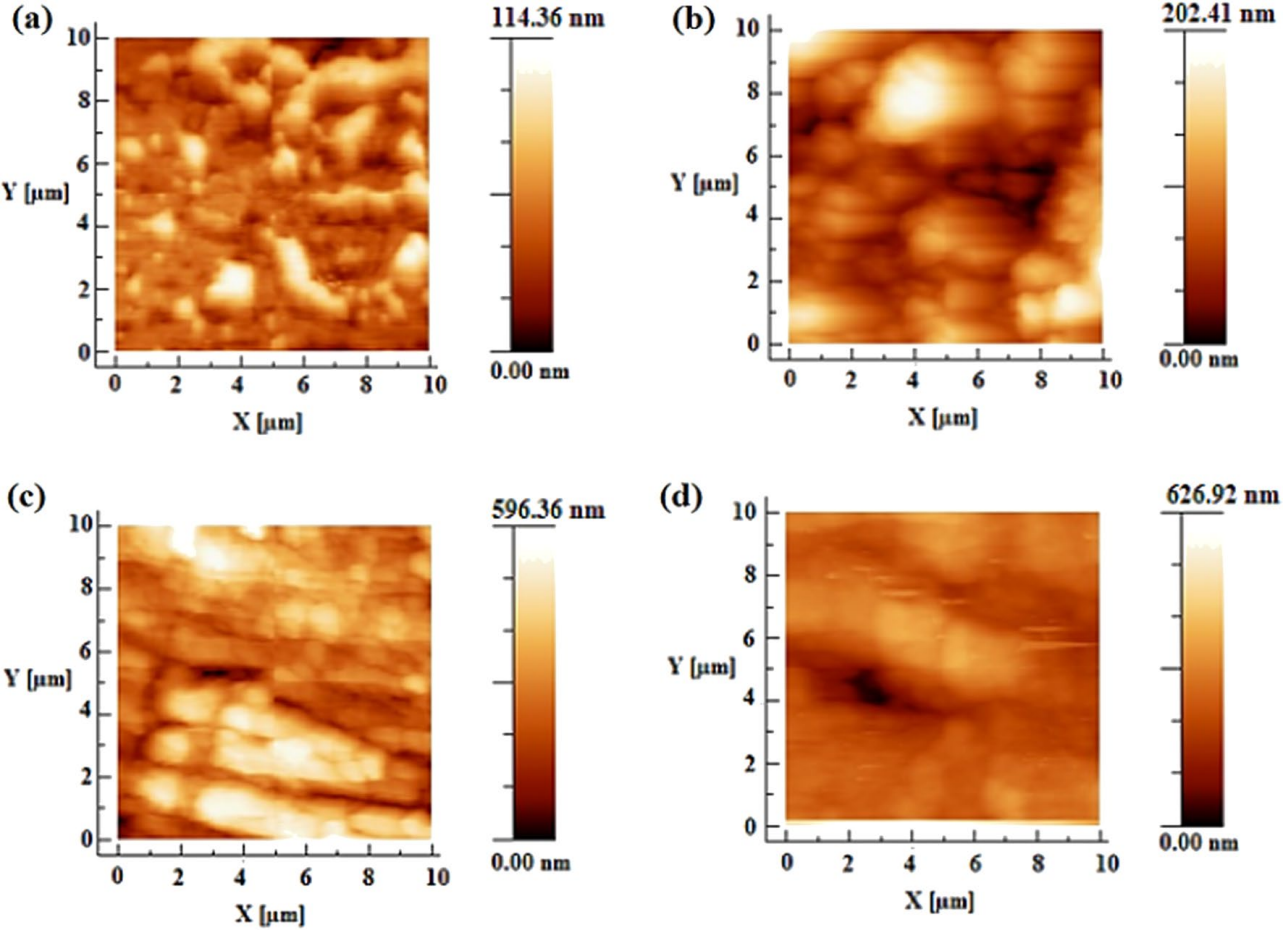
Atomic Force Microscopy showing RMS roughness profile of **a** pure PTFE, and **b**–**d** PTFE-NiO heat-treated nanofiber filter mats of 2, 4, and 6 wt % loadings

Surface roughness of heat-treated pure PTFE and composite PTFE-NiO NFMs containing 2, 4 and 6 wt% NiO loadings was investigated through AFM. Figure 3 shows the comparative root mean surface (RMS) roughness profile of both pure PTFE and composite PTFE-NiO NFMs. Roughness of pure PTFE NFMs was found to be 114 nm, whereas roughness was 202, 596 and 626 nm for specimens with NiO loadings of 2, 4 and 6 wt%, respectively. These results demonstrate that pure PTFE NFMs have lower roughness than the composites, and the RMS surface roughness increases with the wt% of nickel oxide loaded to the electrospinning solution. Thus, the presence of NiO particles on the surface of fibers increases surface roughness, which enhances the hydrophobicity of NFMs [42].

### FTIR analysis

**Fig. 4:**
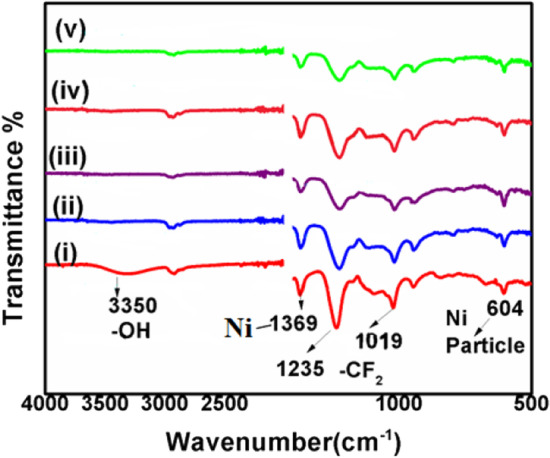
FTIR spectra of (i) as-spun nanofiber filter mats (PTFE-PVA- Ni) and (ii–v) heat-treated PTFE-NiO with NiO loading 2, 4, 6 wt%

Functional groups of both as-spun PTFE-PVA- Ni and heat-treated PTFE-NiO composite NFMs were identified by FTIR analysis and are shown in Fig. 4. The bands at 1019, 1235 cm^−1^ correspond to the characteristic CF_2_ stretching of PTFE, and the band at 3350 cm^−1^ is assigned to the H–OH stretching, which corresponds to the stretching vibrations of PVA [43]. Bands at 604 and 1369 cm^−1^ belong to nickel oxide particles [44]. After heating at 280 °C for 5 min, the characteristic peaks of PVA disappeared completely, while the bands associated with the stretching of –CF2 bonds and those belonging to nickel oxide particles were well-preserved in heated samples.

### Raman spectroscopy analysis

**Fig. 5:**
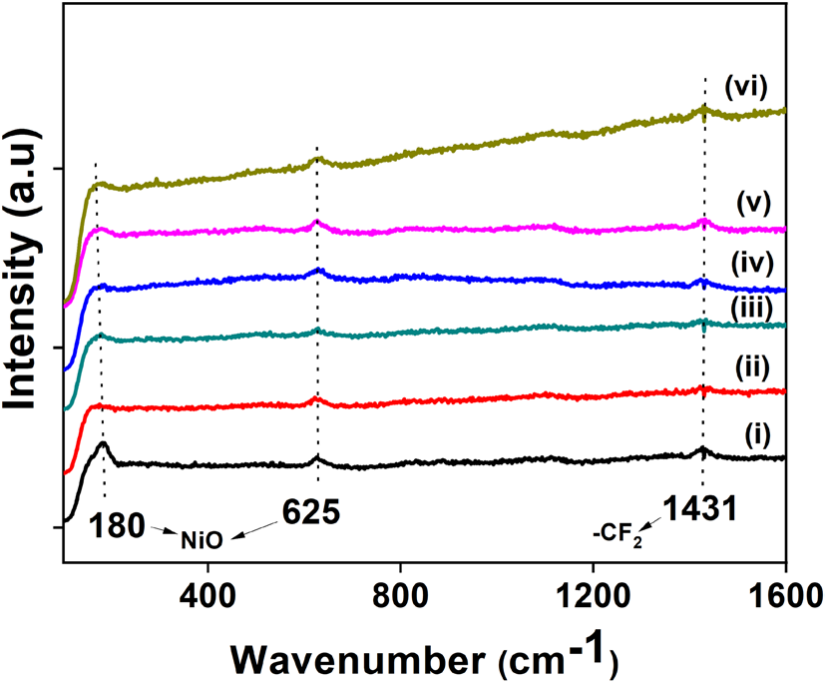
Raman spectra of nanofiber filter mats of PTFE-PVA-Ni as-spun (i–iii) and heat-treated PTFE-NiO samples (iv–vi) for all compositions

Raman spectra of both as-spun PTFE-PVA-Ni and heat-treated NFMs of PTFE-NiO are shown in Fig. 5. Two Raman peaks for nickel oxide are located at about 570 and 1100 cm^−1^ in the range of 200–2000 cm^−1^ in the literature, but in this study the peaks shifted toward 625 cm^−1^. Peaks observed less than 600 cm^−1^ are attributable to first-order scattering one-phonon (1P) longitudinal optical (LO) modes, and the others with the wavelength more than 600 cm^−1^ are owing to second-order scattering two-phonon (2P) 2LO modes of nickel oxide [45]. NiO nanofibers spectra showed forbidden phonon scattering, and the peaks are assigned to 1-phonon scattering (180 cm^−1^), which are not present in nickel oxide single crystal [15]. Lattice defects and the non-stoichiometry in oxygen composition are the major reason behind the phonon scattering phenomena; it lowers symmetry around atoms involved in the formation of phonons in single crystals. The intense peak at 625 cm^−1^ is initiated after the defects [13] as shown in Fig. 5. NiO is an anti-ferromagnetic material. It has two types of magnons, one with spins up and the other with spins down. The magnons nature primarily depends on the structure of NiO at ground state [46]. Raman bands at 1382 cm^−1^ are characteristics of PTFE, which are recognized as symmetric stretching vibrations of A1class CF_2_ polymer in the literature [43]. Here, the Raman bands were observed at 1431 cm^−1^ which are associated with E1 Raman active band due to stretching of CF_2_ bonds. Moreover, the peaks intensification increased with the calcination temperature which may be attributed to the grain growth and densification of fibers in the mats [13, 46].

### Hydrophobic characterization of nanofibers

**Fig. 6:**
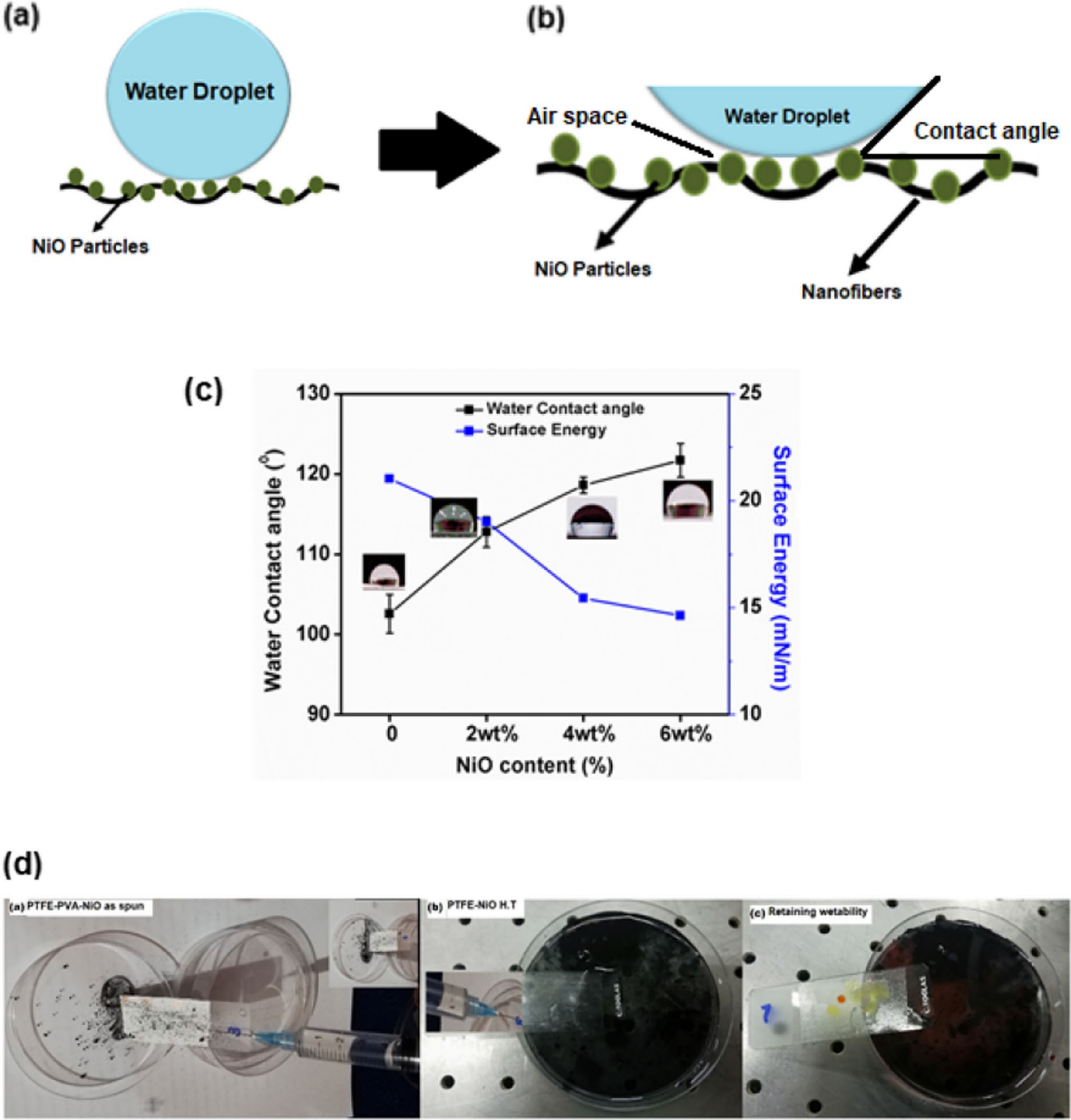
**a**–**b** Schematic illustration of water droplet behavior on nanofiber filter mats, **c** Water contact angle and surface free energy of heat-treated PTFE-NiO nanofiber filter mats, **d** self-cleaning ability of spun PTFE-PVA-Ni and PTFE-NiO heat-treated nanofiber filter mats

A surface is classified as hydrophilic or hydrophobic by how it interacts with water droplet. If a water droplet has a contact angle greater than 90° with the surface, it is called hydrophobic, or if less than 90°, it is designated as hydrophilic [47]. The higher the contact angle, the better the hydrophobicity. This is important for fiber composite mats to avoid the adverse effects of moisture on polar VOC adsorption. [26] The PTFE polymer possesses very low SFE (0.019 N/m)^19^ that gives high hydrophobic property to the PTFE-based composite NFMs. Higher hydrophobicity as clearly indicated in Fig. 6c also results in the migration behavior of components in the heterogeneous electrospun blend [48]. In case of present study, nickel oxide having low molecular weight moves toward the surface and resultantly minimizes surface energy of the polymer mats that helps in efficient adsorption of AAHs. Schematic illustration of water droplet behavior on the surface of heat-treated PTFE-NiO NFMs is shown in Fig. 6a; it endorses the existence of air spaces on these hydrophobic surfaces, which is due to their structural behavior and presence of plenty of bulges at the surface. Since both high surface roughness and high adhesion lead to more voids that make it difficult for water droplets to diffuse across the surface, resultantly the surface of the nanofiber filter mat becomes highly hydrophobic [48]. In contrast, a smooth surface (pure PTFE) with relatively low surface roughness cannot effectively prevent water droplets from entering the surface voids, resulting in the (WCA) the water contact angle value decreases as depicted in Fig. 6a–b. [49] The argument is clarified in favor of PTFE-NiO NFM (higher RMS value, lower surface energy) which exhibits higher hydrophobicity, making it a good candidate for VOC filter application.

The WCA and overall surface energy of heat-treated PTFE-NiO NFMs are shown in Fig. 6c and Table 2. The WCA of the heat-treated control PTFE nanofiber mat was 102.56° ± 2.4, whereas WCAs of heat-treated PTFE-NiO composite NFMs greatly increased from 112.78° ± 1.9 to 121.55° ± 2.2 with an increasing NiO loading from 2–6 wt%. This significant increase in WCA (from pure PTFE to PTFE-NiO 2–6 wt%) was a result of heat treatment that not only removed the hydrophilic carrier polymer PVA from the matrix but also consolidated the porous structure of nano-fibers by densification of PTFE grains in fibers [9] eventually. Both effects led to enhancement of hydrophobicity of heat-treated mats. In addition, it was also observed that WCA increased with an increasing NiO loading in the electrospun mats due to increased surface roughness of NFMs.

**Table 2:** Water contact angle, surface free energy and porosity of heat-treated PTFE-NiO composite nanofiber filter mats

Sample composition	Water contact angle (WCA) (Heat-treated)	Surface Energy (mN/m)	Porosity (%)
PTFE control	102.56° ± 2.40	21.03	50.21
PTFE-NiO (2wt%)	112.78° ± 1.90	19.01	67.40
PTFE-NiO (4 wt%)	119.14° ± 1.00	15.44	69.82
PTFE-NiO (6 wt%)	121.55° ± 2.20	14.63	77.36

Calculated surface free energy of water droplet on fibrous surface is shown in Fig. 6c. Surface free energy of NFMs decreases with an increase in water contact angle from 21.03 mN/m ± 2.14 of pure PTFE samples to 14.63 mN/m ± 3.01 for heat-treated PTFE-NiO samples with different weight content of NiO particles, as shown in Fig. 6c. The low surface free energy values infer that most of the liquid interactions with NFM surface can be related to the dispersive part that mostly includes Van der Waals weak forces [36, 37]. Higher hydrophobicity of NFMs also facilitates filters rejecting dust, moisture content and dismays the irretrievable attachment along with growth of microbes on the surface of air filters [48].

The self-cleaning tests are shown in Fig. 6d for both as-spun PTFE-PVA-Ni and heat-treated PTFE-NiO NFMs. Carbon black was sprinkled on the surface of samples and was then cleaned by flowing water with the aid of syringe. The as-spun NFMs remained wet and got clogged with carbon black particles. In contrast, the heat-treated PTFE-NiO mat surface became rapidly cleaner, stayed dry and retained the hydrophobicity represented by red dot of liquid as shown in Fig. 6d. The above results demonstrate that the surface of the heat-treated PTFE-NiO electrospun NFMs possesses excellent hydrophobicity, self-cleaning properties and extended reusability. These results are in-line with the hydrophobicity and self-cleaning results analysis of Huang [50].

The effect of relative humidity on Electrospun Hydrophobic PTFE-NiO Composite has been investigated in several studies. It has been found that the hydrophobicity of the material increases with an increase in relative humidity. This is because the presence of moisture on the surface of the material reduces the contact angle and makes the material more hydrophilic. The reduction in contact angle reduces the water-repellent properties of the material, which can affect its performance in applications such as filtration. In a study [62], Electrospun Hydrophobic PTFE-NiO Composite was found to have a contact angle of 146° at 20% relative humidity, which increased to 159° at 80% relative humidity. The increase in contact angle at higher relative humidity indicates that the material becomes more hydrophobic with increasing humidity. Additionally, the thickness of Electrospun Hydrophobic PTFE-NiO Composite can also have an impact on its properties and performance. Thicker mats tend to have higher mechanical strength and stiffness, while thinner mats have higher surface area and can offer better filtration performance. In another study [63], the effect of thickness on the filtration performance of Electrospun Hydrophobic PTFE-NiO Composite was investigated. The study found that thinner mats with a thickness of 6 μm had higher filtration efficiency compared to thicker mats with a thickness of 15 μm. The higher surface area of the thinner mats allowed for better particle capture and retention, leading to better filtration performance. Moreover, the study found that thicker mats with a thickness of 30 μm had higher tensile strength compared to thinner mats with a thickness of 10 μm. The higher tensile strength of the thicker mats was attributed to the higher fiber density and interconnectivity of the fibers within the mat [47–50, 62, 63]. Nanofiber composite filter mats typically have high air permeability due to the ultrafine fibers used in their construction, which create small pores that allow air to pass through easily. The air permeability of nanofiber composite filter mats can range from a few liters per minute per square meter (LPM/m^2^) to several hundred LPM/m^2^, depending on the specific material and construction. The pressure drop of a nanofiber composite filter mat depends on the thickness of the filter and the density of the nanofibers used in its construction. In general, nanofiber composite filter mats have a low pressure drop due to their high air permeability. The pressure drop of nanofiber composite filter mats can range from a few Pascals (Pa) to a few hundred Pa, depending on the specific material and construction.

The previous studies [10, 16, 26, 64] also demonstrate that the fabrication of modified electrospun nanofiber mats exhibits high filtration efficiency, good mechanical properties, greater separation efficacy, high adsorption capacity and good reusability and stability.

### AAHs adsorption experiment

Results of adsorption of acetone, toluene and formaldehyde by synthesized heat-treated PTFE mats without metal oxide doping and PTFE-NiO (2-6wt %) are shown in Fig. 8a–c. Adsorption capacity and selectivity of PTFE-NiO nanofiber mats against the tested AAHs depends on the following possible reasons: The interaction of building blocks of PTFE-NiO nanofiber mats with the VOC compounds (Physi-/chemisorption), ionization potential of AAHs, molecular weight, polarity, fiber morphological properties, principal fiber diameter (FD), internal porous structure, pore radius and existence of nickel oxide (functional groups) on the surface of filter mats NFMs [4, 51–54]

AAHs adsorption by composite NFMs is attributed to three steps. Firstly, AAHs molecules adsorb in the grooves or bulges that appear on the surface of fiber during heat treatment. Secondly, adsorption takes place in the internal pores of NFMs through Brownian motion, diffusion and van Edward force (micro pore filling/ capillary condensation) [4, 51, 54, 55]. The large surface specific area (SSA) and well-developed internal pore structure have constructive effect on physisorption [55].

**Fig. 7:**
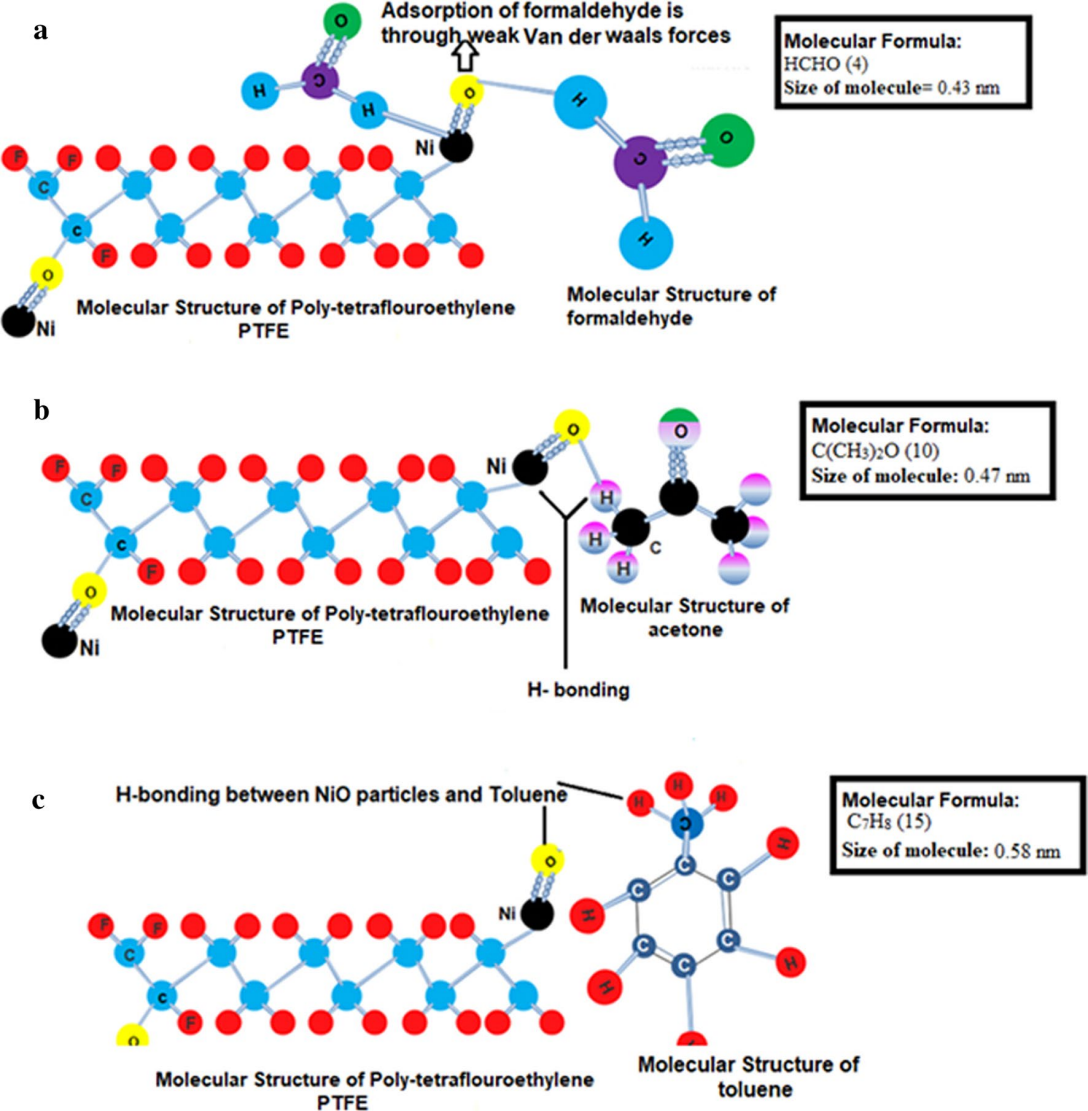
Mechanism of adsorption of AAHs (**a**: formaldehyde,** b**: acetone and** c**: toluene) on the surface of heat-treated PTFE-NiO composite nanofiber filter mats by intermolecular interaction [37]

In the third and last step, VOC molecules adsorbed on the PTFE-NiO NFMs surface with characteristic intermolecular Van der Waals forces, H-bonding, inclusion complexes, dipole–dipole interaction [38, 51, 55] Fig. 7a–c, presence of surface functional groups and the reactive sites on the filter mats surface derive from the defect positions [10, 11]. The results manifest that AAHs molecules adsorption on heat-treated pure PTFE NFMs was deficient. Nonetheless, with the growth of nickel oxide concentration % in these filter mats, there is an increasing trend of AAHs adsorption on the surfaces of PTFE-NiO mats.

Ionization potential (IP), which is a measure of the strength of electronic bonding to the nucleus, is another factor behind effective AAHs adsorption on nanofiber mats surface NFMs. AAHs having smaller IP are more easily adsorbed because their low IP stimulates the development of charge transfer complexes. Accordingly, AAHs have high dispersibility and oxidation efficiency, which ultimately lead to increased molecular trapping of AAHs and increased surface contact with the nanofiber filter mat. The IPs of formaldehyde, acetone and toluene 10.88, 9.71 and 8.82 eV, respectively [56, 57].

**Fig. 8:**
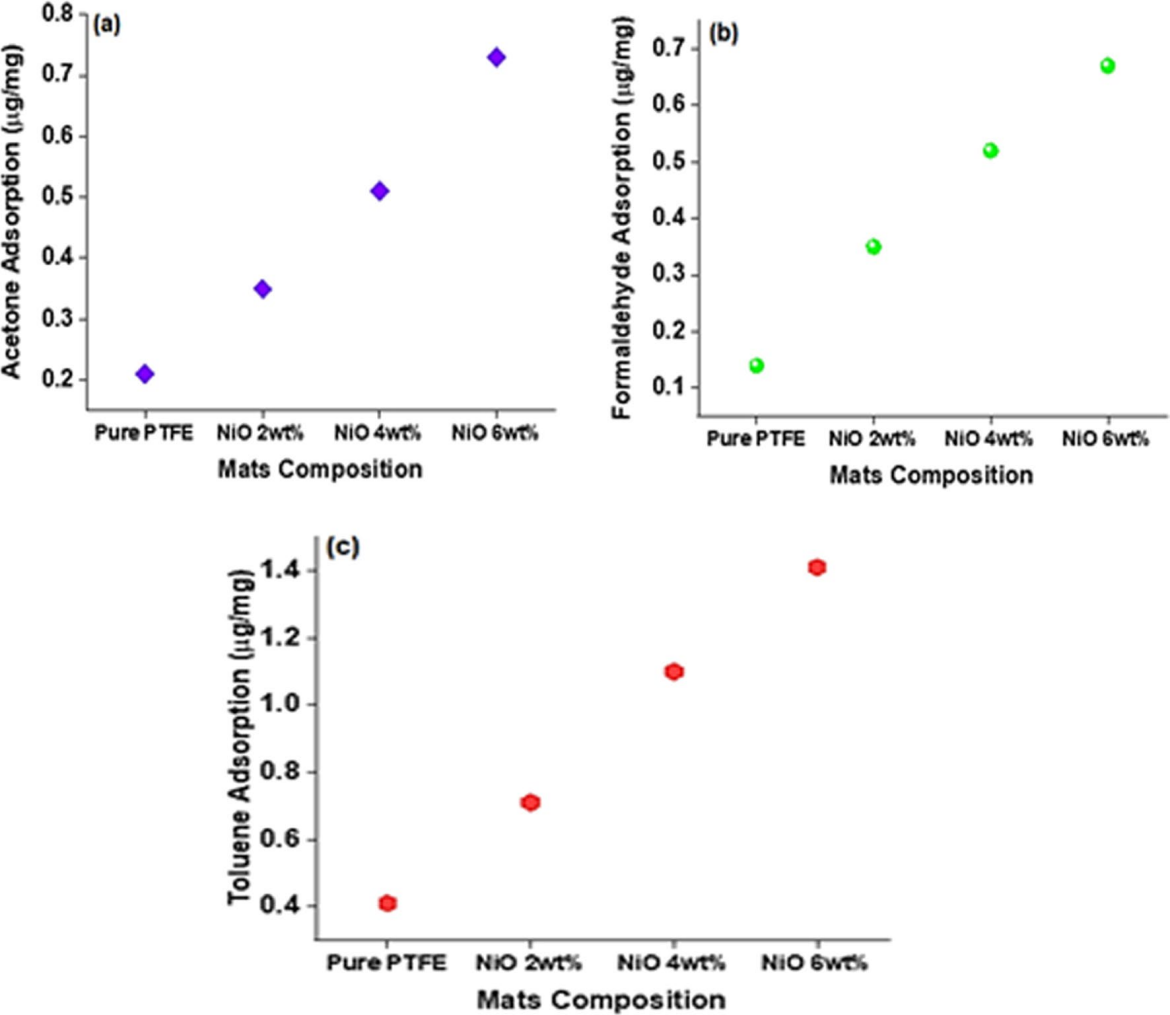
Isotherm of Acetone (**a**), Formaldehyde (**b**) and Toluene (**c**) at different NiO concentrations (µg/mg) determined by UV–Vis spectroscopy

Adsorption capacity of AAHs in the internal porous structure of NFMs as analyzed through UV spectroscopy, and results of AAHs adsorption are shown in Fig. 8a–c. The isotherms of pure PTFE filter mats were 0.41, 0.21 and 0.14 μg/mg of AAHs of toluene, acetone and formaldehyde, respectively, whereas those for filter mats containing 2, 4 and 6 wt% NiO increased to 0.71, 0.35, 0.36 μg/mg for acetone, 1.10, 0.51 and 0.52 μg/mg for formaldehyde, and 1.41, 0.73 and 0.67 μg/mg for toluene, respectively, as shown in Fig. 8a–c. Pure PTFE filter mats adsorbed AAHs in the following order formaldehyde > acetone > toluene. High values of adsorption by formaldehyde on the surface of filter mats are possibly for three reasons: firstly, due to the formation of inclusion complexes between formaldehyde and hydrophobic surface of mat [39, 47], secondly, due to maximum percentage of porosity of mats (Table 2, Figure S3), and lastly due to the presence of some polar group on the surface of mat, residual behind during heat treatment assist in formaldehyde adsorption [58]. In case of PTFE-NiO composite filter mats with different concentrations of nickel oxide, 6 wt% showed best results regarding adsorption of all model AAHs. The isotherm trend of 6 wt% PTFE-NiO composite filter mats is as follows: toluene > acetone > formaldehyde.

Adsorption of somewhat aromatic compounds on the surface of composite fibers occurs by forming π-complex. The high adsorption of acetone by PTFE-NiO 6 wt% was 0.73 μg/mg; for acetone, this is due to its aromatic nature that helps formation of H-bonding with nickel oxide nanoparticles as shown in Fig. 7b. These results show the similar trend of adsorption as reported in various studies [49–51]. It is important to mention here that SEM micrograph shows unique porous morphology for PTFE-NiO 6wt% composite NFMs as shown in Fig. 2d1–d2. This unique morphology further enhances the porosity (Figure S3) and efficient physical adsorption of acetone. This study shows the higher adsorption of acetone compared to the results of Huang (0.582 μg/mg) [26]. Chemisorption of AAHs is enhanced by the presence of functional surface groups of porous materials. Reactive sites on the filter mat surface originate from defect sites, which are in the form of unsaturated atoms at the edges of the basal plane [10]. Surface chemistry of the sorbents is controlled through raw material and modification techniques. Among common surface functional groups, oxygen- and nitrogen-containing groups are the most important species for surface chemisorption [10].

In the porous material, the most common functional groups are oxygen-containing groups, which are further divided into acidic, neutral and basic functional groups. Lewis acid–base pairing can also explain the intermolecular interaction between AAHs and the surface metal ions. NiO single crystal structure with 2LO phonon modes has been reported to show higher oxygen vacant sites; for that reason, NiO can be considered as Lewis acid [58]. Oxygen vacant sites in the lattice of NiO are considered as trapping sites for pollutants (AAHs) in the present case thereby acting as Lewis base [58].

Most adsorbents are non-polar; however, their surface polarity can be enhanced through the O_2_ containing surface functional groups. These functional groups have a preference to adsorb polar AAHs such as acetone, ethanol and methanol through the formation of H- bonds, as shown in Fig. 7b, c for acetone and toluene in the present study. The adsorption capacities of polar compounds are affected by the amount of oxygen containing groups [55].

As reported by Demir [58], physio-sorption of AAHs on the surface of heat-treated PTFE-NiO NFMs generally could be of both polar and non-polar. After heat treatment of nanofiber mats, the surface of nanofiber mats is covered with polar oxide compounds that may effectively coordinate with polar organic compounds such as acetone and formaldehyde in the present case as given in Fig. 7a–b. In polar interaction between VOC molecules and NiO molecules, the possible nearest bond (already calculated on the basis of bond energy) [61] is among oxygen atom of NiO and hydrogen atom of AAHs on the surface. As it is already mentioned in Fig. 6, NiO has two photon on scattering modes (1LO, 2LO) formed due to the presence of lattice defects. Sometimes, these defects in crystal structure of metal oxide during heat treatment are beneficial to the improvement of sensitivity of AAHs [13]. In the present study, these defects proved beneficial in adsorption of AAHs by nanofiber mats as depicted in Fig. 8.

Toluene is generally more distorted due to its aromatic ring electron donor and having a large binding energy. The stability of the inclusion complex is increased by the presence of a methyl group of toluene [52]. Huang tested adsorption of toluene, benzene and chloroform through Polyurethane/Loess powder composite film by GC–MS [1]. However, in the present study, the best possible reason behind maximum toluene adsorption by composite filter mats is increase in porosity 77% (Table 2), decrease in fiber diameter (0.12 µm) and maximum pore radius as indicated in Fig. 2d2 and Figure S3, and large molecular size with more number of atoms involved in molecular interaction as shown in Fig. 7c. [53] The possible reason behind good adsorption capacity of the toluene suggested that it was absorbed by fibers pores, and not simply adsorbed on the fiber surfaces.

Nugraha et al. [59] validated that benzene, toluene and xylene (BTX) adsorption on metal oxide surface is indeed a physisorption state that do not originate from the orbital interaction between BTX and metal oxide surface. Instead, the bonding occurs due to contribution of Van der Waals forces and H-bonding between the surface of metal oxide and the AAHs molecule. This weak interaction has been reported to be caused by the donation of 0.09–0.1 e^−^ to the metal oxide surface from BTX molecules. However, H-atom in toluene (H_toluene_) can easily undergo formation of H-bonding, with oxygen atom present on the surface of metal oxide (O_surf_). Thus, toluene is adsorbed due to H-bonding between O_surf_ and H_toluene_ as shown in Fig. 7c. The present study also showed high amount of toluene adsorption by the filter mats; the reason might be due to bigger molecular radii of toluene and more numbers of atoms involved in interaction in adsorption mechanism as shown in Fig. 7. Another reason for improved AAHs adsorption on the surfaces of synthesized NFMs was increased surface roughness (nm) with the concentration of NiO particles which are given in Fig. 4. Bonding energy (BE) value of toluene on NiO surface calculated by Nugraha [59] was − 1.17 eV. The value of BE will be positive by the intermolecular interaction of compound`s one atom involvement. While the negative bond length value indicates strong adsorption energy between the adsorbed molecules (H) and the surface of NiO, this statement is also in line with present UV adsorption results as shown in Fig. 8a–c

Similarly, the weaker adsorption of formaldehyde on the surface of filter mats, in comparison with acetone and toluene, can be explained by its adsorption mechanism, including weak Van der Waals forces/host guest complexation, small radii of formaldehyde as given in Fig. 7a, and its non-aromatic nature. Formaldehyde lacks formation of inclusion complexes, and its high ionization potential of 10.88 eV causes decrease in adsorption of formaldehyde by composite filter mats [38, 51, 56, 57].

## Conclusions

In this study, NiO-doped PTFE NFMs were fabricated by green electrospinning. Doping of NiO nanoparticles and heat treatment process improved the physio-chemical properties of the PTFE-NiO NFMs for application in AAHs adsorption. In comparison with pure PTFE nanofibers, PTFE-NiO NFMs functionalized with nickel nitrate hexahydrate in the electrospinning blend improved conductivity of the spinning solution, which resulted in fiber formation with finer diameter (0.12 µm), high RMS values (624 nm), enhanced hydrophobicity (121.55°) and high porosity (77.36%). These improved characteristics resulted in enhanced adsorption of AAHs on the surface and in the pores of composite nanofibers filter mats. The composite nanofibers filter mats functionalized by doping 6 wt% NiO nanoparticles were able to have enhanced adsorption of toluene, acetone, and formaldehyde (1.41, 0.73 and 0.67 μg/mg) compared to PTFE mats. The possible mechanisms contributing toward the higher AAHs adsorption are physio-sorption (fiber surface), weak forces (Van der Waals), H-bonding, host guest molecular complexes, molecular radii, ionization potential, crystal defects in nanoparticles and internal core pores adsorption. Owing to their enhanced VOC adsorption capacity, the synthesized composite NFMs may find potential applications for VOC adsorption in various pragmatic sectors.

## Supplementary Information

Supplementary file

## Data Availability

Not Applicable in this section.
