# Supplementary material for: Efficient adsorption of aromatic and aliphatic hydrocarbons by electrospun hydrophobic PTFE-NiO composite nanofiber filter mats
Source: Discov Nano. 2023 Apr 20;18(1):65. doi: 10.1186/s11671-023-03834-4 (PMC10409971; doi:10.1186/s11671-023-03834-4)
Supplement: Supplementary file 1 — Supplementary file [file 11671_2023_3834_MOESM1_ESM.docx]

**Supplementary Information**


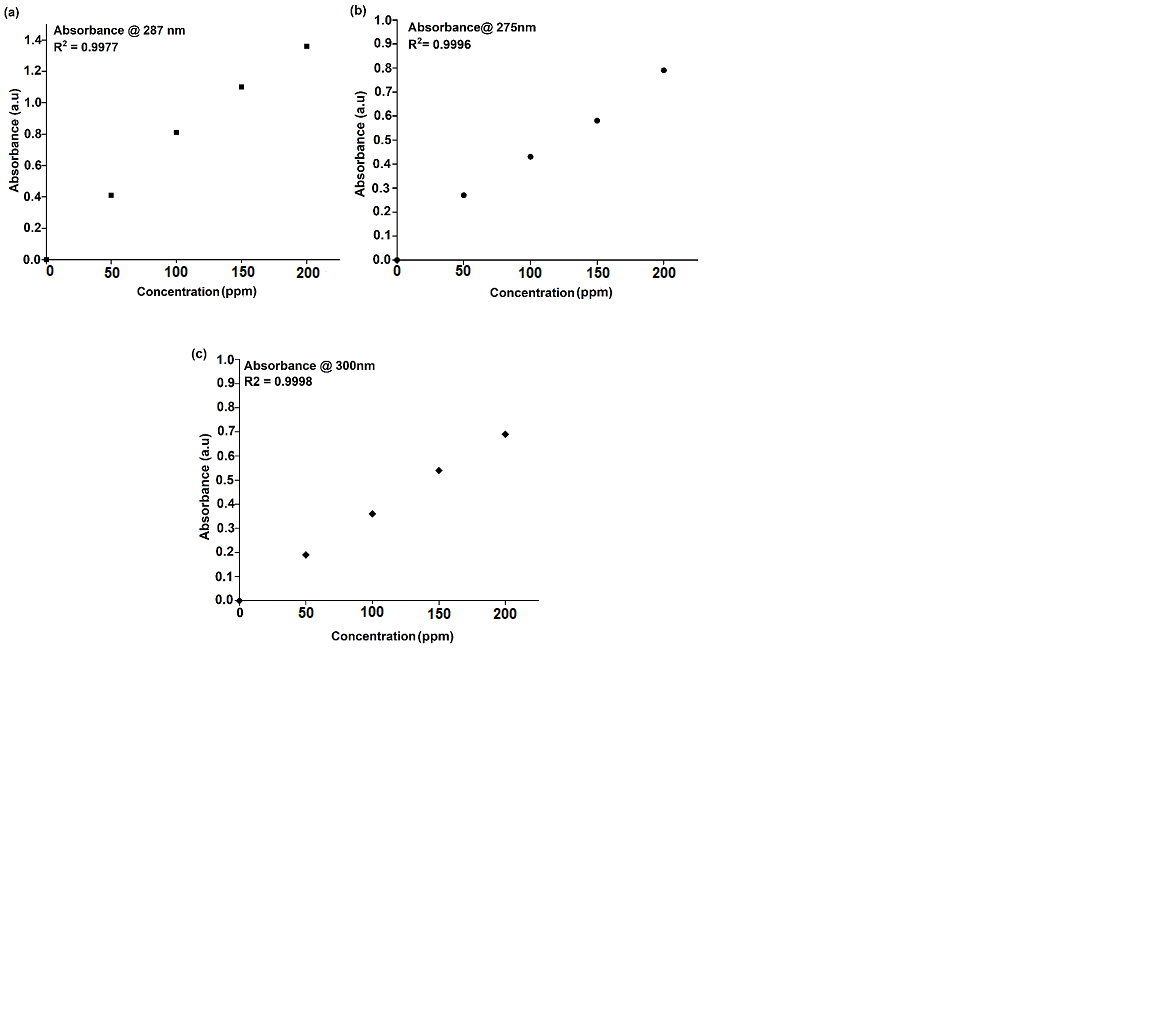


**Figure S1:** Standard calibration curves of model AAHs at different solvent concentrations (b-c). Correlation was found at an absorbance of 300, 287 and 275 nm, respectively, for formaldehyde, toluene and acetone using UV-Vis spectroscopy.


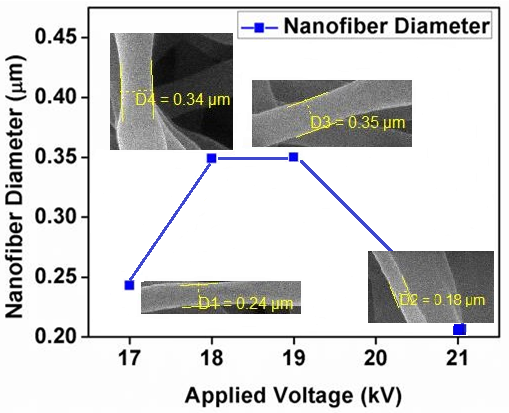


**(a)**


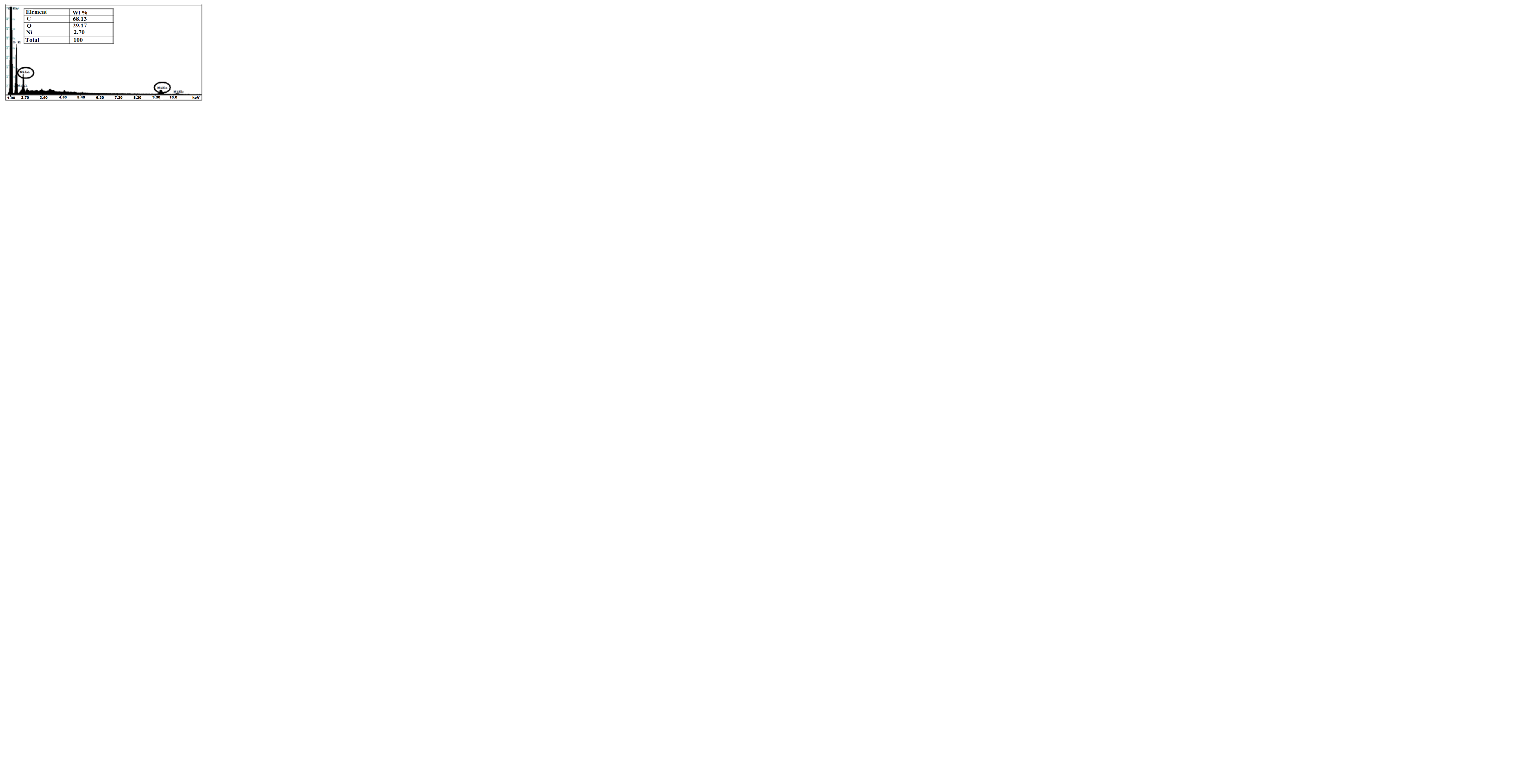


**(b)**

**Figure S2: (a)** Variation in the average fiber diameter of as spun PTFE-PVA-Ni composite nanofiber filter mats as a function of electrospinning voltage, (b) EDS spectrum of PTFE-NiO Nanofiber filter mat


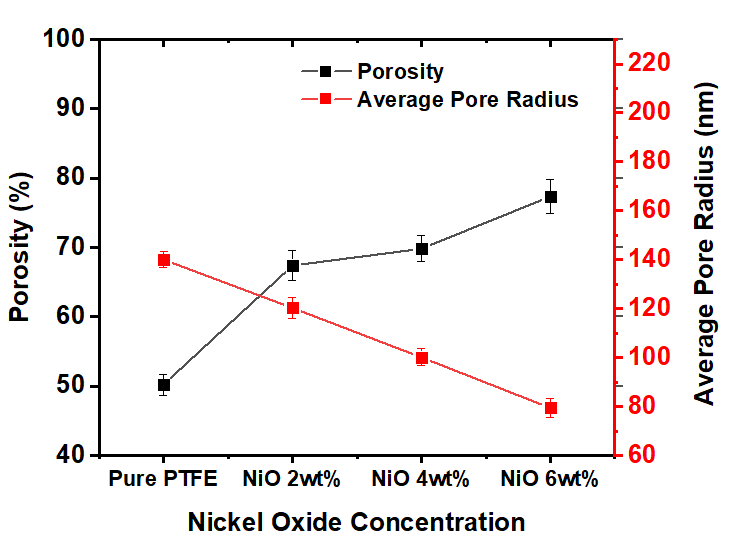


**Figure S3:** Porosity and mean pore radius of heat treated PTFE-NiO 2-6wt% composite filter mats.
